# The effect of telenursing on self-efficacy in patients with non-alcoholic fatty liver disease: a randomized controlled clinical trial

**Published:** 2017

**Authors:** Sorur Javanmardifard, Fariba Ghodsbin, Mohammad Javad Kaviani, Iran Jahanbin

**Affiliations:** 1 *School of Nursing and Midwifery, Ahvaz Jundishapur University of Medical Sciences, Ahvaz, Iran*; 2 *Community Based Psychiatric Care Research Center, Shiraz Geriatric Research Center, School of Nursing and Midwifery, Shiraz University of Medical Sciences, Shiraz, Iran*; 3 *Gastroenterohepatology Research Center, School of Medicine, Shiraz University of Medical Sciences, Shiraz, Iran*; 4 *Department of Community Health Nursing, School of Nursing and Midwifery, Shiraz University of Medical Sciences, Shiraz, Iran*

**Keywords:** Non-alcoholic fatty liver, Diet, Physical activity, Telenursing, Self-efficacy

## Abstract

**Aim:**

This study aimed to assess the effect of telenursing on nutritional behavior and physical activity self-efficacy in patients with non-alcoholic fatty liver disease (NAFLD).

**Background:**

NAFLD is the most common liver disorder, which has a chronic course. Therefore, routine monitoring of these patients by medical staff helps them actively participate in the healing process and promote their self-efficacy.

**Methods:**

In this randomized controlled clinical trial, 60 patients were chosen through convenience sampling among patients with NAFLD. After obtaining written informed consents, the participants were randomly divided into an intervention and a control group (each containing 30 subjects). The participants received diet consultation individually and were taught how to perform physical activities. Telephone intervention was conducted in the intervention group for 12 weeks. The study questionnaires were completed by the participants before and after the intervention. The data were analyzed using the SPSS statistical software.

**Results:**

Based on the results, the mean score of nutritional behavior and physical activity self-efficacy increased in the study groups after the intervention. This increase was statistically significant only in the intervention group. Additionally, the two groups were significantly different regarding the mean scores of nutritional behavior and physical activity self-efficacy.

**Conclusion:**

Telenursing could improve self-efficacy and physical activity in patients with NAFLD.

## Introduction

 Non-Alcoholic Fatty Liver Disease (NAFLD) represents a spectrum of clinical and pathological conditions defined by macro vesicular steatosis in the absence of alcohol (1), which can advance to severe conditions such as fibrosis, cirrhosis, liver failure, hepatocellular carcinoma, and finally death (2). In this disease, abnormal accumulation of fat is created in the liver tissue, associated with metabolic syndrome (3), such as obesity, hypertension, diabetes, and impaired fat metabolism (4). The prevalence of this disease is approximately 25-45% in the general population (5). Besides, the peak prevalence of NAFLD has been reported 50-65 years of age, and the spread of the disease is significantly higher in males than in females in the same age group (4). 

Non-pharmacological lifestyle interventions have a positive impact on NAFLD and are recommended as the first line of therapy (6). Several studies have also shown that gradual weight loss along with regular physical activity can help to treat this disease (4,7). Therefore, patients should be encouraged to have a proper diet along with increased physical activity (4).

Generally, timely interventions and regular follow-up are essential in order to promote healthy behaviors. Patients with regular follow-up are more likely to change their unhealthy behaviors (8). Some studies have shown that routine monitoring helps patients and families to actively participate in the healing process and be successful in controlling the disease (9). On the other hand, modern technologies has provided the opportunity to shift treatments from hospitals and clinics to patients’ homes (8). American Nursing Association has considered telenursing as a subset of telemedicine that focuses on providing specific nursing services (10). Telenursing increases patients’ access to influential and effective nursing. Telephone, as an available means of communication, is increasingly used in telenursing. This method of care delivery not only reduces the costs and facilitates access to care services, but it also improves the relationship between patients and caregivers. Today, use of telenursing enables nurses to perform actions such as monitoring, training, collecting data, performing nursing interventions, controlling pain, and providing family support (11).

In general, patients’ self-care is affected by several factors, including self-efficacy, mood, obstacles, and the ability to perform self-care activities, with self-efficacy playing an important role in promoting self-care behaviors (12), self-efficacy is considered to be an important prerequisite to behavior change. Studies have shown that individuals with high self-efficacy showed greater willingness to participate in challenging behaviors, and offered better interpretation about health and well-being (13). Since nutritional behavior self-efficacy and physical activity are important factors in achieving the expected results by patients with NAFLD, designing self-care programs based on patients’ self-efficacy can have an impact on improving their conditions and preventing complications, and hospitalization (14). In the studies conducted by Mohammadi et al. (2017) and Young et al. (2014 ) on patients with diabetes, telenursing follow-up was effective in improving self-efficacy in females with diabetes (15, 16). Behzad et al. also investigated the effect of empowerment program regarding telenursing on self-care behaviors among hypertensive old adults. The results revealed that this program based on telenursing care was effective in promoting self-efficacy in self-care behavior of elderly patients with hypertension (17). Moreover, due to continuing deterioration of NAFLD, by following treatment processes, patients can be treated at early stages and unpleasant complications can be prevented.

Up to now, few researches have evaluated the effect of exercise and diet in patients with NAFLD, and no study has been conducted on the impact of telenursing on nutritional behavior and physical activity self-efficacy in such patients. Thus, the present study aimed to assess the effect of telenursing on self-efficacy in patients with NAFLD.

## Methods

**Diagram 1:**
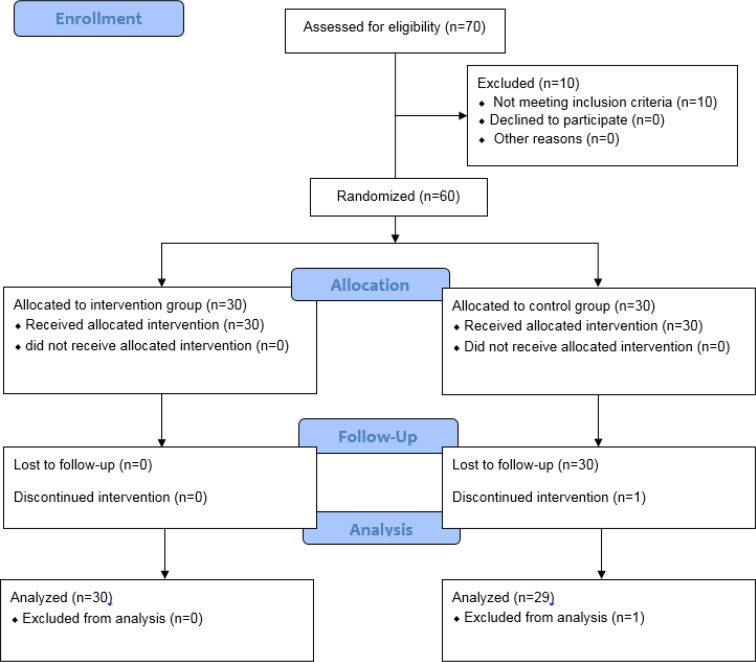
CONSORT Flow Diagram

This randomized controlled clinical trial was conducted from May 2013 to December 2014 (Diagram 1). It was approved by the Ethics Committee of Shiraz University of Medical Sciences (Ethics code: CT-92-92-62-6602) and was registered in the Iranian Registry of Clinical Trials (IRCTcode: IRCT2015040411691N5). According to the results of other studies and using the following formula, 60 patients with NAFLD who had referred to gastroenterology clinics affiliated to Shiraz University of Medical Sciences were recruited into the study (18, 19). It should be noted that one of the participants in the control group was excluded from the study due to lack of cooperation.

$n=\frac{{2\sigma }^{2}{\left(z_{1-\frac{\alpha }{2}}+z_{1}-\beta \right)}^{2}}{{\left(\mu_{1}-\mu_{2}\right)}^{2}}$

(SD_1_=17.5, SD_2_=15, µ_1_=48, µ_2_=36.2, α=0.05, 1-β=0.8)

NAFLD was confirmed by an expert physician using ultrasound and laboratory tests. The inclusion criteria of the study were aging 19 years or above, being overweight or obese (Body Mass Index (BMI) > 25 kg/m2), having the ability to do moderate physical activities, having a telephone at home or a mobile phone, and not having any speech or hearing problems. On the other hand, the exclusion criteria were having the history of chronic liver diseases, such as viral or drug hepatitis, confirmed Wilson disease, and primary hemochromatosis, suffering from hyperthyroidism or hypothyroidism, bile duct cancer, diabetes mellitus, obesity due to excessive use of corticosteroid, Cushing’s syndrome, Addison syndrome, and chronic infections such as tuberculosis, use of hepatotoxic drugs within the past 6 months, incidence of gall stones in the gallbladder, having been exposed to petrochemicals, alcohol consumption, drug abuse, and genetic diseases related to lipid disorders. 

After obtaining written informed consents, simple randomization based on the table of random numbers was used to allocate patients to intervention (n=30) or control (n=30) groups. In doing so, numbers 0-4 were assigned to the control group and numbers 5-9 to the intervention group. At first, the researcher explained about the nature of the disease, factors contributing to the disease, progression and prognosis, complications of the disease, and importance of treatment and follow-up. Then, the researcher asked the participants to complete the demographic information form. 

To determine nutritional behavior self-efficacy, Sherer and Maddux’s (1982) nutritional behavior self-efficacy questionnaire was used. The items of the questionnaire were scored based on a 5-point Likert scale. Accordingly, the minimum and maximum scores of nutritional behavior self-efficacy were 10 and 50, respectively. Then, the scores were divided into two groups of desirable self-efficacy (scores above 25) and undesirable self-efficacy (scores below 25). The reliability and validity of this questionnaire were determined by Hosseinzadeh et al. (2010) in patients with coronary artery disease using internal consistency and content validity methods, respectively. Based on the results, Cronbach’s alpha coefficient was 0.90 for the questionnaire (14). 

Pender’s 8-item questionnaire was used to determine the patients’ physical activity self-efficacy. The items of this questionnaire were scored using a 5-point Likert scale. Accordingly, the minimum and maximum scores of self-efficacy were 8 and 40, respectively, with higher scores representing greater self-efficacy. The reliability and validity of this questionnaire were determined by Aghamolaei et al. (2008) using content validity and internal consistency methods, respectively. Based on the results, Cronbach’s alpha coefficient was 0.87 for the questionnaire (20).

In this study, a nutritionist was hired to offer consultation to all participants. The participants received dietary advice in written. Then, they were asked to do moderate physical activities at least 30 minutes a day 4-5 times a week to raise their heart rate and respiration. Jogging, cycling, aerobic exercises, and any other activities with similar intensity were prescribed, as well. In addition to one-on-one consultation, all the participants received a training booklet. 

In the intervention group, intervention by telephone lasted for 12 weeks in order for the participants to follow up the recommended diet and physical activity. The participants were given self-report forms designed by the researcher to register their daily diets and physical activities. This helped them easily report the items during the telephone follow-ups. The forms included evaluation of the participants’ adherence to diet and physical activities. In case a patient had not followed the diet and training program, the researcher attempted to recognize and analyze the reasons to provide a possible solution. It should be noted that the conversations were recorded by the researcher in each session. The researcher contacted all the patients between 8 AM to 8 PM twice a week during the first month and once a week during the second and third months. Averagely, each conversation lasted for 15-20 minutes (21, 22).

The control group participants did not receive any interventions and were only followed up as usual by a specialist. It is worth mentioning that the control group participants received the educational booklet after the intervention. 

At the end of the 12 weeks, the participants were asked to complete the related questionnaires again. Then, the data were entered into the SPSS statistical software (version 16) and were analyzed using chi-square test, paired t-test, and independent t-test.

## Results

This study was performed on 60 patients (30 in each group). There were 8 females (26.7%) and 22 males (73.3%) in the intervention group and 6 females (20.7%) and 23 males (79.3%) in the control group. There was no significant difference between the two groups concerning gender. In addition, the mean age of the participants was 40.3 and 38.3 years in the intervention and control groups, respectively (Table 1). The results of independent t-test showed no statistically significant difference between the two groups in this regard. Additionally, the results of Fisher’s exact test revealed no significant difference between the two groups with respect to marital status and education level (Table 2). Thus, the two groups were similar with regard to demographic characteristics. 

**Table 1:** Comparison of the two groups regarding mean age

P value	Mean ± SD	Max	Min		
0.437				Age	
	40.30±9.63	64	24	Intervention group	
	38.34±9.53	70	26	Control group	

**Table 2:** Frequency distribution of marital status in the study groups

Control group	Intervention group		
		Marital status	
28 (96.6)	26 (86.7)*	Married	
1 (3.4)	4 (13.3)	Single	

* Number (percent)

According to Table 3, the mean score of nutritional behavior self-efficacy and physical activity increased in both groups after the intervention. However, the results of paired t-test indicated that this increase was significant only in the intervention group (p<0.001).

Based on Table 4, the results of independent t-test showed that the two groups were significantly different with regard to the mean scores of nutritional behavior self-efficacy and physical activity (p<0.001).

According to Table 5, the results of McNemar test demonstrated that change from undesirable to desirable nutritional mode was statistically significant in the intervention group (p<0.001), but not in the control group (p>0.05).

**Table 3:** Comparison of the two groups’ mean scores of nutritional behavior and physical activity self-efficacy before and after the intervention

Self-efficacy				P-value
Nutritional behavior				
	Intervention group			<0.001
		Before the intervention	32.13±7.07	
		After the intervention	44.83±4.95	
	Control group			0.054
		Before the intervention	35.34±5.89	
		After the intervention	36.93±7.08	
Physical activity				
	Intervention group			<0.001
		Before the intervention	20.63±6.47	
		After the intervention	30.50±5.96	
	Control group			0.062
		Before the intervention	21.31±6.67	
		After the intervention	22.27±7.30	

## Discussion

Follow–up is an important part of nursing services. In fact, various methods are used in the healthcare system to follow up patients’ status. Traditional follow-up methods included patients’ referral to care centers, home visits by healthcare providers, etc. In spite of their efficiency, these methods require human resources, time, and high expenses. Therefore, use of telephone follow–up to track patients with chronic diseases has increased in the recent years (23). Using telenursing, patients require continuous monitoring of their abilities to cope with their illnesses and learn how to change their lifestyle. In addition to education, telephone follow-up is established by creating an ongoing dynamic care relationship, which is followed by improving patients’ quality of life, reducing complications, increasing patient satisfaction, and promoting health and quality of services (11). Thus, it can be claimed that telephone follow-up is one of the most cost-effective and efficient follow-up methods in chronic diseases (23). 

Generally, telenursing refers to the implementation of nursing services to patients by telecommunication. By using telephone, nurses can understand patients’ needs and help them meet their demands. This method can reduce patients’ stress, anxiety, and depression, increase their self-esteem, and transfer patient care from clinics and hospitals to patients’ homes (24). Several studies have proved the effectiveness of nursing follow-up by phone in achieving the stated objectives. In this context, Zakerimoghadam et al. conducted a study on the impact of telenursing on adherence to diet in patients with type 2 diabetes. Their result showed that telenursing led to improvement of adherence to diet in patients with type 2 diabetes (11). 

In another study, Borhani et al. investigated the effect of telenursing on glycemic control and BMI of patients with type 2 diabetes. They concluded that telenursing was able to improve the patients’ metabolic indices (25). 

Surveying and following up patients’ behaviors by telephone at home helps to institutionalize healthy behaviors in the field of disease control by tracking and modification of training materials (26). Evidence suggests that a managing program performed by a nurse along with phone follow-up could be used as a successful and practical treatment method to create changes in patients’ behaviors (27). Parchami Iraqi et al. conducted a study on the effect of telephone counselling on quality of life of patients with colostomy. Based on the results, telephone counselling was effective in the patients’ physical, mental, and social aspects (24,28). Similar studies have also come to similar results about the positive impact of telenursing on adherence to diet (8, 9, 29).

According to the results presented in Table 3, the mean score of nutritional behavior self-efficacy and physical activity increased in the study groups after the intervention. However, the results of paired t-test showed that this increase was statistically significant only in the intervention group. In addition, the results of independent t-test presented in Table 4 showed a significant difference between the two groups regarding the mean scores of nutritional behavior and physical activity self-efficacy. These results reflected the positive impact of telenursing on improving nutritional behavior and physical activity self-efficacy in the intervention group after the intervention. These results were in line with the study by Boroumand et al., which confirmed that telenursing was an appropriate method for promotion of cardiac self-efficacy in patients with coronary artery diseases (23).

**Table 4:** Comparison of the two groups’ mean differences of nutritional behavior and physical activity self-efficacy scores before and after the intervention

Self-efficacy			
Nutritional behavior		Physical activity	
Intervention group	Control group	Intervention group	Control group
Mean± SD	Mean± SD	Mean± SD	Mean± SD
12.70±5.99	1.59±4.25	9.87±4.45	0.96±2.68
P-value**=**<0.001		P-value**=**<0.001	

* p<0.001

**Table 5:** The desirability status of nutritional behavior self-efficacy scores in the study groups before and after the intervention

Nutritional behavior self-efficacy		Before the intervention	After the intervention	P-value
		Frequency (percent)	Frequency (percent)	
Intervention group	Desirable condition	24(80)	30(100)	0.031
	Non-desirable condition	6(20)	0(0)	
Control group	Desirable condition	29(100)	28(96.55)	1.000
	Non-desirable condition	0(0)	1(3.45)	

* (p>0.05)

Self-efficacy is an important part of fundamental human behavior. Humans have little incentives to behave unless they believe that desirable results obtained by their actions will be achieved. Thus, it is imperative to point out that self-efficacy should be strengthened to increase the probability of healthy behaviors (30). On the other hand, understanding factors such as self-efficacy can prevent unhealthy habits and behaviors in the presence of other factors (31). According to Navidian et al. Bandura believed that individuals’ evaluation of their ability in a particular situation severely affected their decision on the type of activities, and insist on their continuation. Considering the fact that self-efficacy implies individuals’ beliefs and confidence about their own abilities in showcasing a specific behavior even in a tempting situation, it can be claimed that self-efficacy is effective in weight control by avoiding eating during positive and negative emotional situations, easy access to food, social pressures from others, and physical discomfort (32). Bas et al. investigated the relationship between self-efficacy and weight loss in overweight men and women, and came up with the conclusion that high nutritional behavior self-efficacy scores were correlated to weight loss and weight control (33). Similarly, Liou et al. evaluated the impact of self-efficacy on compliance with low-fat diet and showed that self-efficacy was significantly linked to a healthy diet (34). Moreover, multiple studies have indicated that structures such as self-efficacy, benefits, perceived barriers, and interpersonal support, were effective in changing physical activity behaviors (32). In this context, Rejeski et al. conducted a research on the relationship between weight loss and nutritional behavior self-efficacy, and reported that increasing physical activity could increase individuals’ self-efficacy in controlling nutritional behaviors. In addition to consuming energy, it could also help weight loss through strengthening the level of self-efficacy as a mediating variable (35). Moreover, Didarlu et al. assessed physical activity in women with diabetes based on the theory of developed reasoned action. The results demonstrated that among the factors influencing physical activity such as awareness of the disease, personal beliefs, abstract norms, perceived self-efficacy, and behavioral intention, perceived self-efficacy was the strongest and most effective variable in the desire to do physical activities (36). Furthermore, Roozbahani et al. carried out a research on the association between self-efficacy and stages of change and women’s physical activity behavior after giving birth. They found that self-efficacy played an important role in physical activity behavior. They also argued that verbal encouragement from consultants and healthcare providers by providing appropriate feedback in the field of physical activity was effective in improving self-efficacy (37). 

Based on Table 5, before the intervention, 24 patients in the intervention group had desirable conditions and 6 had undesirable conditions in terms of nutritional behavior self-efficacy. However, in the control group all the participants had desirable conditions in terms of nutritional behavior self-efficacy. After the intervention, the patients who had undesirable conditions in terms of nutritional behavior self-efficacy in the intervention group had developed desirable conditions. However, one patient in the control group shifted his nutritional behavior condition from desirable to undesirable. Based on the results of McNemar test, change from undesirable to desirable condition was statistically significant in the intervention group, but not in the control group. 

It seems that in order to increase patients’ trust in following a healthy diet and improve nutritional changes in the long run, it is essential to understand the specific aspects of the disease, and its treatment methods. Hence, patients who believe that following a healthy diet will improve their conditions will have higher confidence in managing changes occurred in their diet. Hosseinzadeh et al. aimed to predict nutritional behavior self-efficacy in patients with coronary artery disease. Their results showed that increasing patients’ understanding of the disease led to a 1.05-fold increase in the chance of nutritional behavior self-efficacy. Therefore, they concluded that provision of appropriate training and continuous follow-up in the field of the disease and its treatment was effective in increasing disease awareness and promoting self-efficacy (14).

Overall, telenursing can be effective in improving patients’ self-efficacy by providing support, verbal encouragement, and giving proper feedback to patients in the field of physical activity and compliance to the diet. As mentioned previously, in order to increase the probability of healthy behaviors, self-efficacy must be strengthened. In order to do this in the long run, the training needs to be repeated by medical staff, especially nurses. 

The study findings indicates that telenursing could improve the patients’ self-efficacy in adherence to diet, physical activity, and healthy behaviors associated with the disease by increasing their awareness. Essentially, regular follow-up and provision of consultation services by telephone after clinical consultation can maintain and complete the effect of the received training, stabilize healthy behaviors, and improve treatment outcomes. Further studies are recommended to investigate the effect of complementary medicine on improvement of patients with NAFLD. Conducting qualitative researches on these patients would be beneficial, as well.

## Conflict of interests

The authors declare that they have no conflict of interest.
